# An exploratory approach to identify microRNAs as circulatory biomarker candidates for epilepsy-associated psychiatric comorbidities in an electrical post-status epilepticus model

**DOI:** 10.1038/s41598-023-31017-9

**Published:** 2023-03-20

**Authors:** Eva-Lotta von Rüden, Heike Janssen-Peters, Maria Reiber, Roelof Maarten van Dijk, Ke Xiao, Isabel Seiffert, Ines Koska, Christina Hubl, Thomas Thum, Heidrun Potschka

**Affiliations:** 1 Institute of Pharmacology, Toxicology, and Pharmacy, Ludwig-Maximilians-University (LMU) Munich, Koeniginstr. 16, 80539 Munich, Germany; 2 Hannover Medical School (MHH), Institute of Molecular and Translational Therapeutic Strategies (IMTTS), Hannover, Germany

**Keywords:** Neuroscience, Biomarkers

## Abstract

Patients with epilepsy have a high risk of developing psychiatric comorbidities, and there is a particular need for early detection of these comorbidities. Here, in an exploratory, hypothesis-generating approach, we aimed to identify microRNAs as potential circulatory biomarkers for epilepsy-associated psychiatric comorbidities across different rat models of epilepsy. The identification of distress-associated biomarkers can also contribute to animal welfare assessment. MicroRNA expression profiles were analyzed in blood samples from the electrical post-status epilepticus (SE) model. Preselected microRNAs were correlated with behavioral and biochemical parameters in the electrical post-SE model, followed by quantitative real-time PCR validation in three additional well-described rat models of epilepsy. Six microRNAs (miR-376a, miR-429, miR-494, miR-697, miR-763, miR-1903) were identified showing a positive correlation with weight gain in the early post-insult phase as well as a negative correlation with social interaction, saccharin preference, and plasma BDNF. Real-time PCR validation confirmed miR-203, miR-429, and miR-712 as differentially expressed with miR-429 being upregulated across epilepsy models. While readouts from the electrical post-SE model suggest different microRNA candidates for psychiatric comorbidities, cross-model analysis argues against generalizability across models. Thus, further research is necessary to compare the predictive validity of rodent epilepsy models for detection and management of psychiatric comorbidities.

## Introduction

Epilepsy is a global health care issue affecting 70 million people worldwide and the prevalence of active epilepsy is usually between four and 12 per 1000 people each year^1^. The presence of comorbidities in epilepsy is almost the norm and epilepsy rarely occurs alone: more than 50% of people suffering from epilepsy have additional psychiatric comorbidities^2^. Among these, anxiety and depression have been identified as prevalent and serious comorbidities, which have a major impact on quality of life in patients suffering from epilepsy^3,4^. Therefore, there is a need to detect and manage these comorbidities associated with epilepsy. While great progress has been made in the development of prognostic and diagnostic biomarkers for epilepsy^5^, the development of biomarkers associated with psychiatric comorbidities, such as depression and anxiety disorders, as well as cognitive dysfunction, is still in its infancy^6–8^. However, the vision of targeted treatments for epilepsy-associated comorbidities depends on the development of biomarkers that allow identifying patients at risk to develop psychiatric comorbidities and to further design individually tailored treatment.

Per definition, a biomarker is “a characteristic that is measured as an indicator of normal biologic processes, pathogenic processes, or responses to an exposure or intervention, including therapeutic interventions”^9^. Per definition, microRNAs are a class of evolutionarily highly conserved, small (18–22 nucleotides in length), non-coding, regulatory RNAs^10^ with each microRNA regulating the expression of hundreds of target genes^11^. The identification of circulatory biomarkers might provide a basis for non-invasive approaches^5^, and could substantially improve the identification of people suffering from epilepsy-associated psychiatric comorbidities.

Animal models with induced chronic epilepsy display various neurobehavioral and biochemical alterations and are therefore suitable to assess epilepsy-associated comorbidities including psychiatric disorders like depression or anxiety^12,13^. In this context, the selection of an appropriate animal model should also consider the assessment of the severity and burden on the animals. As part of a research consortium^14^, we have made relevant progress in identifying and validating parameters that form a basis for evidence-based severity assessment and reached a new level of sensitivity^15–19^. Improved sensitivity of severity assessment approaches may be of particular importance for animal welfare-based prioritization of models and validation of refinement measures. Besides the data from various behavioral assessments, we have also included the concentration of brain-derived neurotropic factor (BDNF) from serum samples of the animals in this study. There is growing evidence that the modulation of BDNF in the human brain may play a vital role in the development of depression^20–24^. In particular, BDNF levels tended to be lower in serum samples of patients diagnosed with depression^20^, and could be increased with antidepressant treatment^22^.

The aim of the present exploratory and hypothesis-generating study was to identify circulating microRNAs as potential blood biomarkers with consistent differential expression across different rat models of epilepsy (amygdala kindling with focal/generalized seizures, chemical and electrical induced status epilepticus (SE) with the development of spontaneous recurrent seizures). Recent evidence suggests, for instance, a potential role for miR-132 as a general distress-associated biomarker in murine models for gastrointestinal diseases^25^. These microRNA candidates should be evaluated as potential biomarkers for identifying patients at risk to develop epilepsy-associated psychiatric comorbidities, and, in addition, to assess animal distress and cumulative severity in epilepsy models.

## Results

### TaqMan ArrayCard microRNA screening

**Figure 1:**
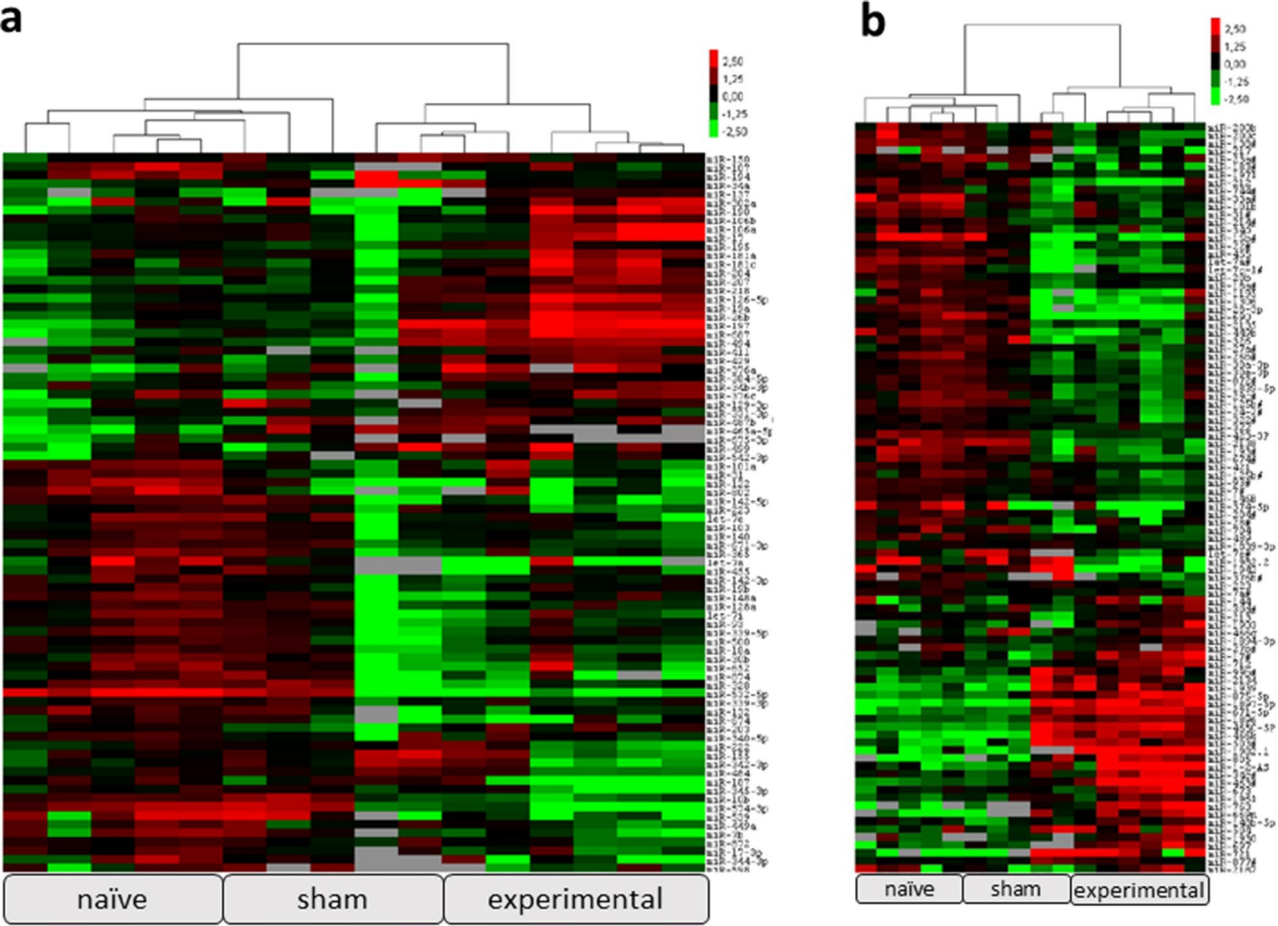
MicroRNA screening of 750 microRNAs. (**a**) rodent card A and (**b**) rodent card B. Heat maps representing up- (red) and down-regulation (green) of microRNAs in naïve control (n = 5), sham animals (n = 5) and animals with SE (n = 6). Comparison of animals with SE to sham animals revealed 48 significantly up- or down-regulated microRNAs with a minimum Ct-value of 30 in all samples. The heatmap was generated with Cluster3.0 (https://www.encodeproject.org/software/cluster/). cluster3 original website is http://bonsai.hgc.jp/~mdehoon/software/cluster/, original paper is https://pubmed.ncbi.nlm.nih.gov/14871861/

Screening of 750 candidate microRNAs (Fig. 1) revealed 48 significantly up- or down-regulated microRNAs in experimental animals (n = 6) compared to sham animals (n = 5) with a minimum Ct-value of 30 in all samples^25–27^.

### Filtering and selection of microRNA candidates

Candidate microRNAs (n = 750) were subsequently subjected to a statistical validation procedure comprising the following steps (an overview is provided in Supplementary Fig. 1): first, 311 candidate microRNAs were removed (n = 439), because the expression assay revealed that the microRNA is not expressed in any of the samples (n = 166, median Ct-value according to Livak and Schmittgen^27^) or due to too many missing values to do a *t* test (n = 145). Next, 383 candidate microRNAs that were not significant in the *t* test (experimental cohort vs. sham cohort; p > 0.05) were removed (n = 56). 7 candidate microRNAs with a fold change between − 1 and 1, which may indicate no relevant increase or decrease of expression levels, were subsequently removed (n = 49). For the remaining candidate microRNAs, the correlation with seizure frequency and duration during the monitoring phase was assessed. Thereby, 35 candidate microRNAs displaying a spearman correlation coefficient higher than 0.5 in combination with a p value < 0.05 with either seizure frequency or duration were removed (n = 14). This was done to exclude any microRNA that might just reflect the intrinsic epilepsy severity, i.e. the density and duration of seizure events. Lastly, 3 candidate microRNAs with a minimum Ct-value higher than 30 were removed (n = 11)^25–27^.

### Principal component analysis (PCA) of behavior and microRNA expression

**Figure 2:**
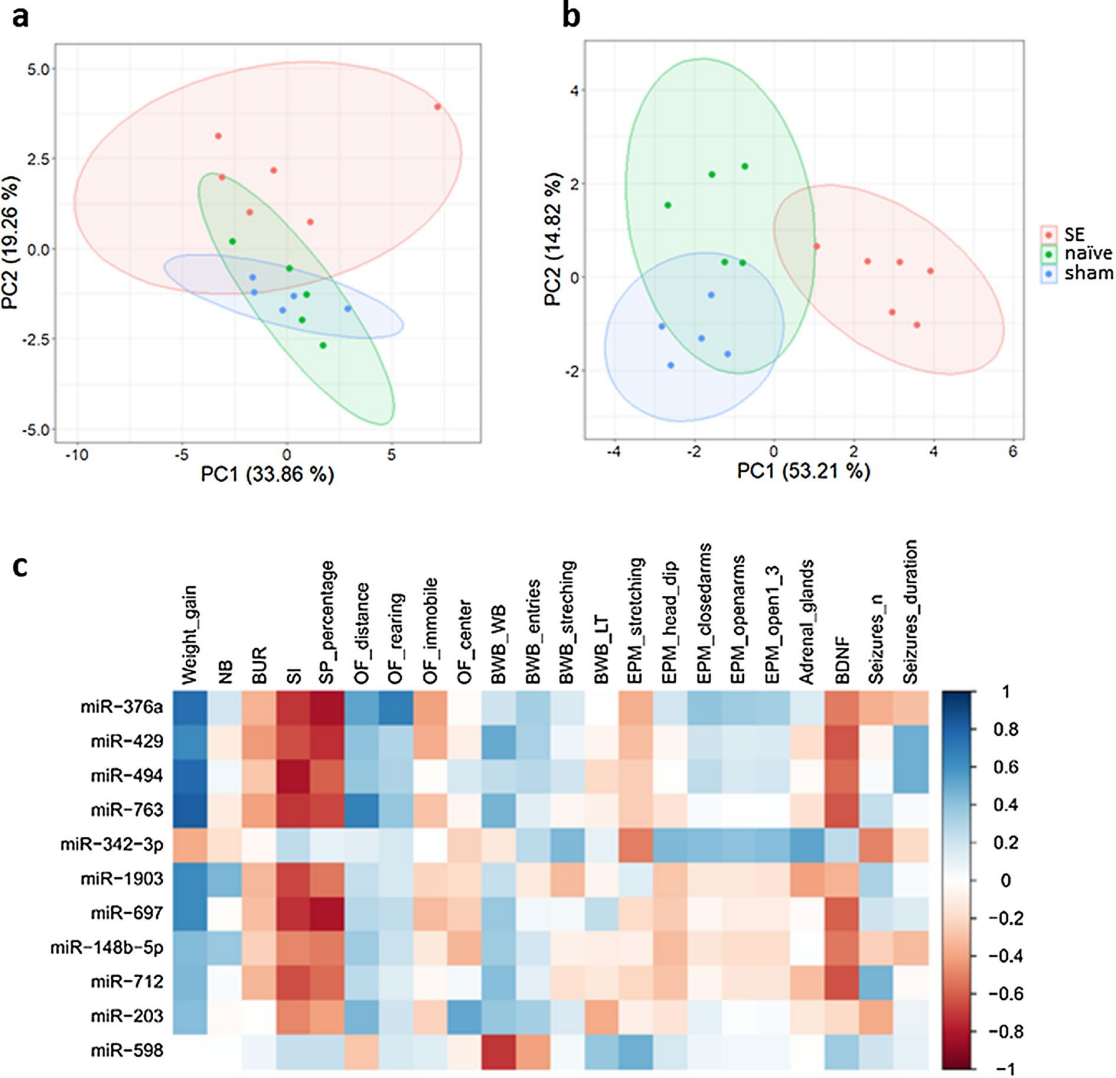
Principal component analysis (PCA) and correlation analysis of selected microRNAs (miR-148b-5p, miR-203, miR-342-3p, miR376a, miR-429, miR-494, miR-598, miR-697, miR-712, miR-763, miR-1903) in the electrical post-SE model. Illustration of the PCA of behavioral and biochemical parameters (**a**) and of expression data from selected microRNAs (**b**). Correlation matrix of the selected microRNAs and behavioral and biochemical parameters (**c**): the heat map illustrates the correlation (Spearman correlation coefficient) between the different parameters. The analysis identified a correlation between six microRNAs and weight gain in the early phase of epileptogenesis (positive correlation, blue) and with social interaction, saccharin preference and BDNF in the phase of epilepsy manifestation (negative correlation, red). All abbreviations are described in the Supplementary file. *naïve* naïve controls (n = 5), *sham* sham controls (n = 5), *SE* experimental animals with seizure history (n = 6), *NB* nestbuilding activity, *BUR* burrowing behavior, *SI* time animals spent in active social interaction, *SP_percentage* saccharin preference, *OF* open field, *OF_distance* open field distance moved in total, *OF_rearing* number of rearing postures in the open field, *OF_immobile* time the animal was immobile in the open field, *OF_center* time the animals spent in the center region of the open field, *BWB* black and white box, *BWB_WB* time the animals spent in the white box, *BWB_entries* number of transitions from black to white compartment, *BWB_stretching* number of stretching postures of the animal, *BWB_LT* latency to the first entry of the black box from the white box, *EPM* elevated plus maze, *EPM_stretching* number of stretching postures, *EPM_head_dip* number of the times the animal looked down from one of the open arms, *EPM_closedarms* time the animal spent in closed arms, *EPM_openarms* time the animal spent in open arms, *EPM_open1_3* time the animal spent in the outer 1/3 of the open arms, *Adrenal_glands* weight of adrenal glands (**g**), *BDNF* brain-derived neurotrophic factor in pg/ml, *Seizures_n* number of seizures, *Seizures_duration* duration of seizures.

Principal component analyses were conducted for behavioral and biochemical parameters as well as for expression data of selected microRNAs (n = 11) obtained in the electrical post-SE model. Data from behavioral assessments complemented with data on weight gain and serum BDNF levels were subjected to principal component analysis (naïve controls (n = 5); sham controls (n = 5), animals with SE and the development of spontaneous recurrent seizures (n = 6); Fig. 2). The analysis indicated a separation of SE animals from naïve control and sham animals along PC2. The individual behavioral data from SE animals suggest a higher inter-individual variance. PCA analysis of the individual microRNA expression values of the eleven selected microRNAs (miR-148b-5p, miR-203, miR-342-3p, miR376a, miR-429, miR-494, miR-598, miR-697, miR-712, miR-763, miR-1903) suggests a clear separation of data from SE animals and of data from naïve and sham controls with comparable inter-individual variance for all groups.

### Correlation matrix between behavior and microRNA candidates

**Figure 3:**
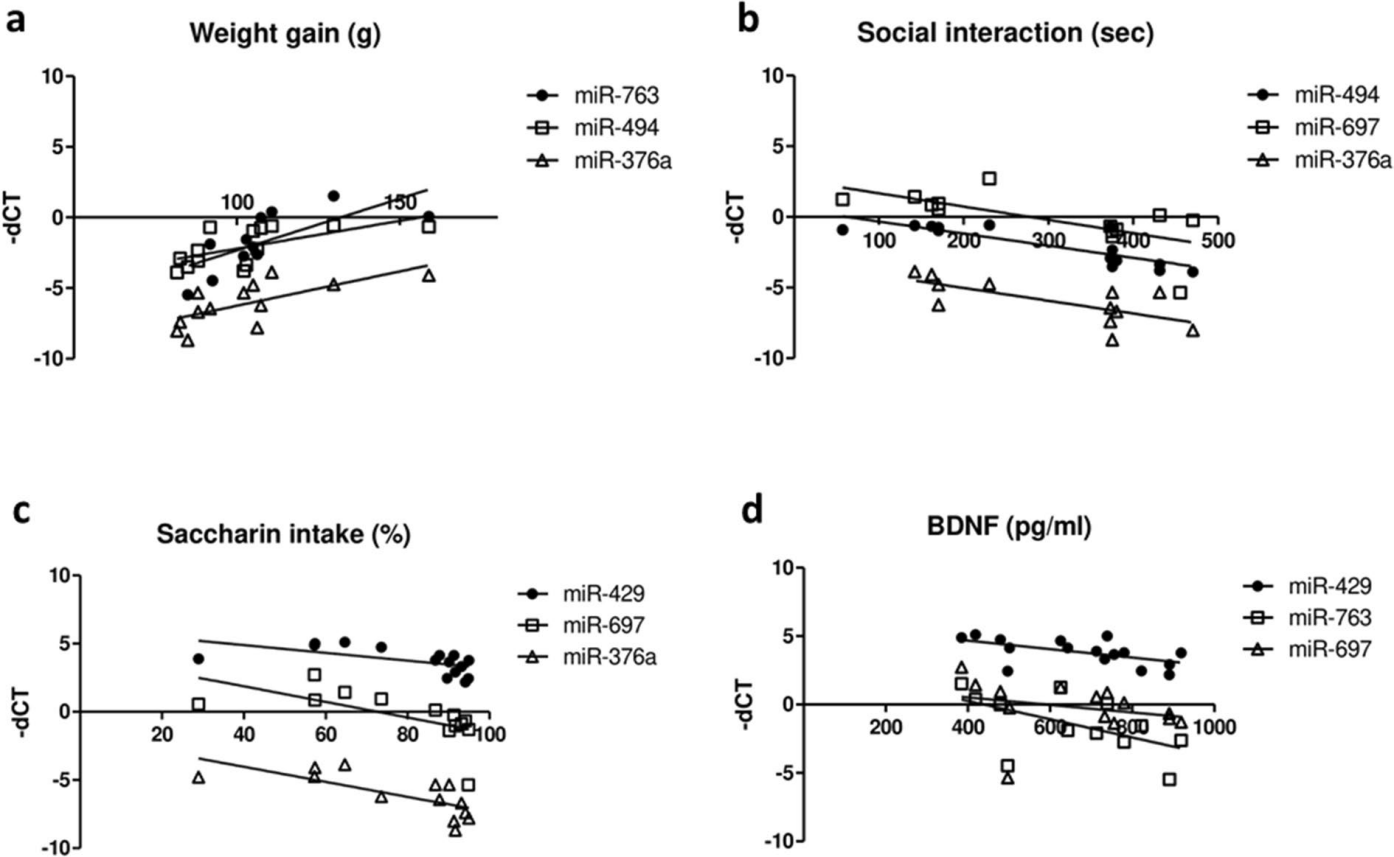
Correlation analysis between three selected microRNA candidates and selected behavioral and biochemical parameters from the electrical post-SE model. Analysis of correlation (Spearman) between the three microRNA candidates (out of eleven) with the strongest positive/negative Spearman correlation coefficient and the following behavioral and biochemical parameters: weight gain (**a**), social interaction (**b**), saccharin preference (**c**), and BDNF expression levels (**d**). *dCT* delta Ct (cycle threshold) value. For each analysis n = 12–16.

A correlation matrix with the spearman correlation coefficients between the microRNA candidates (n = 11) and the various biochemical and behavioral parameters measured in the same animals is shown in Fig. 2. A detailed description of the parameters used for the correlation matrix can be found in Supplementary Table 1. Notable observations are that six out of eleven candidates exhibit a comparable correlation with selected behavioral and biochemical data. These six microRNAs include: miR-376a, miR-429, miR-494, miR-697, miR-763 and miR-1903: the correlation matrix indicates a positive correlation with weight gain and a negative correlation with social interaction (Fig. 3a,b). In addition, the heatmap suggests a negative correlation of miR-376a, miR-429, miR-494, miR-697, and miR-763 with saccharin preference as well as a negative correlation of miR-429, miR-494, miR-697, and miR-763 with plasma BDNF levels (Figs. 2 and 3c,d). Among the microRNAs for which a positive correlation with body weight gain is assumed, heatmap correlation plots also suggest a negative correlation for several of these microRNAs with social interaction, saccharin preference, and BDNF. All correlation values (p-value and correlation coefficient r) are provided in Supplementary Table 2.

### Validation of microRNA candidates using quantitative real-time PCR

**Figure 4:**
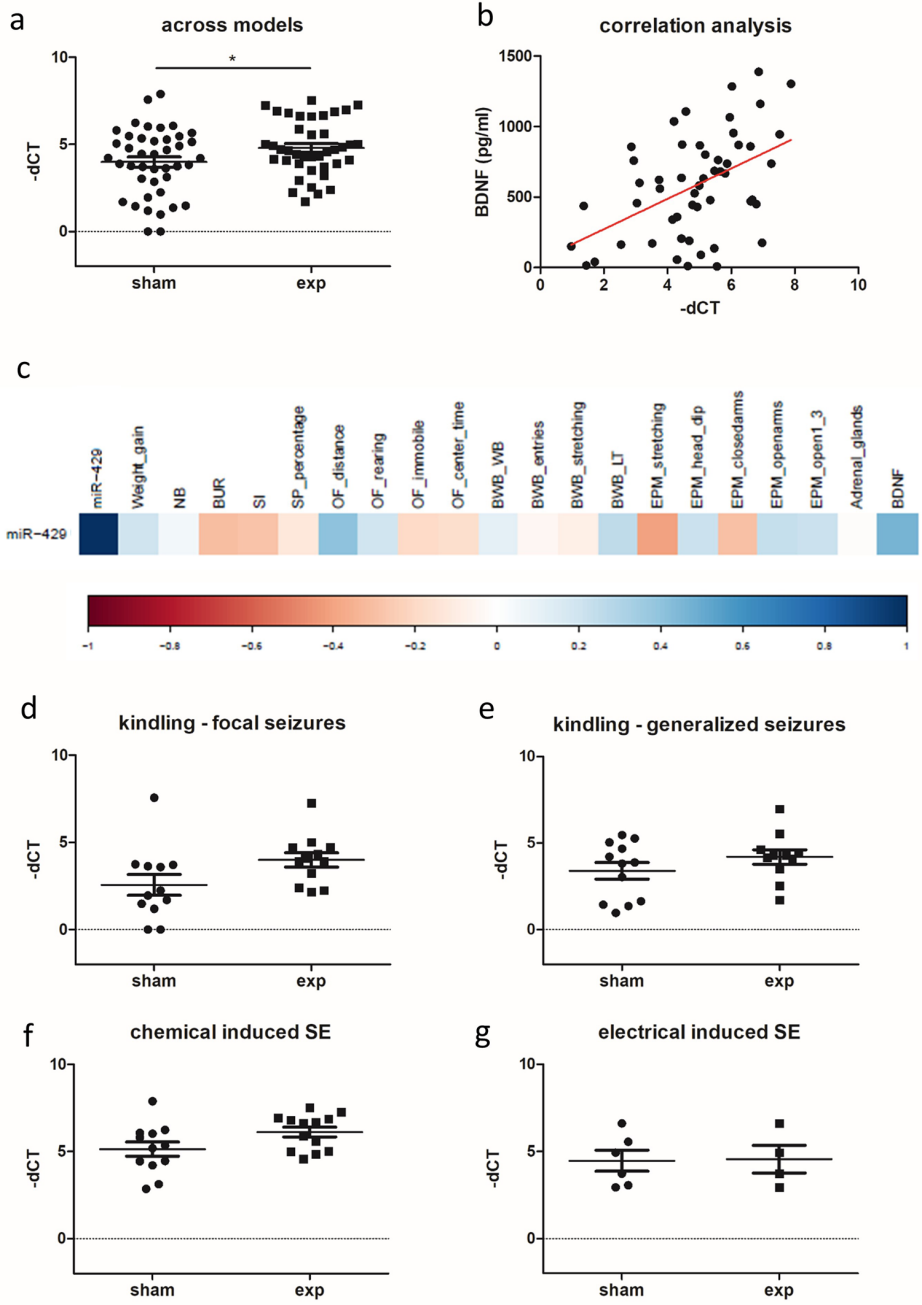
Validation of miR-429 expression in other epilepsy models. (**a**) MicroRNA miR-429 is upregulated in experimental animals (n = 40) when compared to sham animals (n = 42) across models (p = 0.0383). (**b**) Cross-model-correlation analysis of miR-429 expression levels and BDNF concentrations revealed a significantly positive correlation (Spearman correlation). (**c**) Correlation of miR-429 with several behavioral and biochemical parameters across models (positive correlation, blue; negative correlation, red). (**d**) Kindling model with focal seizures (sham/exp n = 12), (**e**) Kindling model with generalized seizures (sham n = 12, exp n = 11). (**f**) Chemical post-SE model (sham n = 12, exp n = 13) and (**g**) electrical post-SE model (sham n = 6, exp n = 4). In the kindling model with focal seizures (**d**) and in the chemical post-SE model (**f**), miR-429 expression levels were not increased in animals with seizure activity (p = 0.0628 and p = 0.0619, respectively; *t* test). *sham* sham controls, *exp* experimental animals with seizure history, *dCT* delta Ct (cycle threshold) value.

The microRNA candidates were further validated in three additional experimental epilepsy models: (1) the kindling model with focal seizures, (2) the kindling model with generalized seizures as well as (3) the chemical post-SE model. Among the eleven pre-selected microRNAs (miR-148b-5p, miR-203, miR-342-3p, miR376a, miR-429, miR-494, miR-598, miR-697, miR-712, miR-763, miR-1903), only three microRNAs were characterized by an expression level allowing differential expression analysis (miR-203, miR-429 and miR-712). Among them, miR-429 was significantly upregulated (p = 0.0383) in experimental animals (n = 40) in comparison to sham animals (n = 41) (Fig. 4). When we analyzed biochemical and behavioral parameters in these models, we only detected a moderate positive correlation between BDNF and miR-429 (FDR-corrected p = 0.01; r = 0.057; Fig. 4). All correlation values (FDR-corrected p-values and correlation coefficient r) are provided in Supplementary Tables 2 and 3. The expression levels of miR-429 have been analyzed in the individual models (Fig. 4). dCT-values did not differ between the different models.

## Discussion

In this study, we could identify several candidate microRNAs significantly upregulated in plasma samples from distinct chronic rat epilepsy models.

Several microRNAs including miR-376a, miR-429, miR-494, miR-697 miR-763, and miR-1903 were identified as interesting biomarker candidates based on findings from the electrical post-SE model. Considering the predictive validity of the different paradigms^28,29^, the negative correlation with social interaction and saccharin preference suggests that these microRNAs may be linked with psychiatric comorbidities including depression and autism. A potential link is further supported by the negative correlation with BDNF. In a chronic rat epilepsy model, low serum BDNF levels have previously been linked with stress vulnerability and a predisposition to behavioral alterations that may recapitulate psychiatric comorbidities in patients^30^.

An increase in anhedonia-associated behavior detected in the saccharin preference test and a decrease in social interaction have previously been suggested as sensitive parameters for evidence-based severity assessment in different rodent models^15–18^. Thus, the correlation between the microRNAs and the behavioral patterns also points to a potential informative value of these circulatory microRNAs for the assessment of severity in rodent models.

Considering the potential link between the pre-selected microRNAs and the behavioral changes in the electrical post-SE model, it is of particular interest to further explore the potential of these pre-selected microRNAs as biomarker candidates for psychiatric comorbidities on one hand, and severity in laboratory rodents on the other hand, and to explore the generalizability of these results. The results from the PCA show that the entity of microRNAs that had successfully passed the validation procedure are suitable to distinguish between individual animal data from the epileptic cohort and the control group.

For this purpose, we have selected two different chronic epilepsy models including electrical kindling with repeated electrically induced seizures and a chemical post-SE model. In the cross-model analysis considering data from all models, miR-429 was standing out as the only molecule with an upregulation in experimental rats versus electrode-implanted rats. To our knowledge a regulation of miR-429 has not yet been reported in epileptic tissue from rodent models or human patients. However, it has been identified as a promising candidate biomarker especially in the field of cancer research, and, as a member of the miR-200 family, it is involved in the regulation of epithelial to mesenchymal transition, which can be considered as a crucial step in tumor metastasis^31^. At the first glimpse our findings might indicate a potential value as a biomarker for psychiatric comorbidities in patients and severity in laboratory animals. However, the subsequent correlation analysis did not confirm a consistent cross-correlation of the microRNA with behavioral and biochemical alterations in the different chronic epilepsy models used.

In this context, it needs to be considered that the kindling paradigm with the common exposure to repeated stimulations does not result in spontaneous seizures^32^. Thus, while the models used for comparison in this study share the generation of a hyperexcitable network, the kindling paradigm commonly lacks the formation of a network and epileptic focus that spontaneously triggers recurrent seizures^32,33^. Moreover, in comparison with models with spontaneous recurrent seizures, behavioral and biochemical alterations in this model are rather subtle and mild^15–18,34^. Concerning a direct comparison between the electrical and chemical post-SE model, we and others have previously reported relevant differences in behavioral patterns and molecular alterations. In this context, it should be considered that exposure to the convulsant can be associated with compound-related effects^32,35^. In addition, it is well known that the neuropathology of the pilocarpine model is rather extreme often exceeding respective pathological alterations in patients with temporal lobe epilepsy^32,36^.

Taken together our molecular findings further confirm substantial differences between the chronic models. Thus, it will be of particular relevance to invest more time and efforts to explore and compare the predictive validity of the models when it comes to behavioral alterations as a basis for translational research in the context of psychiatric comorbidities.

As this is an exploratory, hypothesis-generating study to identify potential circulatory biomarker candidates for epilepsy-associated psychiatric comorbidities, we are aware that we have to avoid overinterpretation, and that some limitations of the experimental design have to be considered. When further evaluating the significance of the findings in this study, it also has to be considered that blood sampling represents an invasive means, which was carried out at the end of the experiments. In addition, time points of blood sampling (36 h after the last seizure, between 9:00 and 10:30 a.m.) can affect the outcome since microRNA expression patterns can be regulated following the circadian rhythm of rats. Besides, there is a general question of specificity, in particular, concern exists that the increased microRNA levels detected in circulatory fluids originate from damage in brain tissue^37^. Considering the entire filtering and selection process for microRNA candidates with the sequential steps for validation, it needs to be emphasized that this is an exploratory process, in which optimal quantity and quality of samples cannot always be guaranteed. On the one hand, the conduction of 439 comparisons (experimental cohort vs. sham cohort) for expression analyses without post-hoc tests as a first filtering step increases the risk of inflated type I error rates. On the other hand, the correction of p values afterwards to mitigate false discovery rates would have carried the risk of excluding promising candidates, which can still be filtered and validated in subsequent steps. Considering the exclusion of microRNAs with a fold change between − 1 and 1, it needs to be emphasized that microRNAs with small fold changes are not necessarily irrelevant to the exploratory research question^38^. So far, however, no standard method exists on normalization, preprocessing, and downstream analyses of microRNA expression patterns^39^. An appropriate normalization technique, however, should strengthen analytical rigor by minimizing technical and experimental bias without introducing noise^39,40^. In the present study, for qRT-PCR data, in particular for microRNA relative expression assay, we used a classical ddCT approach according to Livak and Schmittgen^27^, taking median Ct-values as normalizer to perform a global normalization of each array card. Moreover, in order to enhance specificity, it is necessary to define criteria that make it possible to exclude microRNAs whose expression is altered only by the intrinsic severity of epilepsy itself. However, the positive correlation of a candidate microRNA with duration and/or frequency of seizures, which was considered an exclusion criterion in the validation process, can reduce the sensitivity of the candidate search. More precisely, there is convincing evidence that seizure frequency in patients with epilepsy can correlate with depression and anxiety disorders in a relevant manner (e.g.^41–43^). As a consequence of the last validation step, the removal of microRNAs with Ct-values > 30 increased the risk of missing CNS-derived microRNAs whose intrinsic concentration is low, which should be considered a limitation in the context of sensitivity. For all models used in this study, female animals have been selected, as the mortality of male rats in the electrical post-status-epilepticus model is quite high^44^. When using male rats, the required number of animals must be dramatically increased to obtain reliable data. From an ethical point of view, this is concerning. In addition, the NIH called for a gender balance and the inclusion of female animals in preclinical studies, as male animals usually are overrepresented^45^. Furthermore, based on the estrous cycle, we expect a higher variance in female rats, and on the long-term we want to identify robust microRNA expression alterations. Thus, we decided to use female rats in this first exploratory study. However, in the future it will be of interest to conduct a comparative study in male rats.

With respect to translational validity, it needs to be considered that the extrapolation from animal behavioral and/or biochemical data to a patient’s clinical disease phenotype is a conceptual issue^46–48^. In particular, the level to which preclinical data may be extrapolated from animal models to human disorders, and, more specifically, the association of animal behavioral alterations with human psychiatric comorbidities, requires further validation. Moreover, the biopsychosocial model, as proposed by Engel^49^, remains particularly applicable in the field of clinical psychiatry due to the complexity and polymorphism of the clinical phenotype of psychiatric diseases^50^. Therefore, animal models of depression are generally considered less ‘valid’ with respect to the three criteria (1) predictive, (2) construct, and (3) face validity^51^. While the latter can be enhanced in a relevant manner, predictive and construct validity of animal models in psychiatry research remain a controversially discussed issue^46,51–53^. Finally, with regard to the exploratory nature of the first part of the study, the interpretation and conclusions of the results must be made with caution: the study presents an approach for a first, not fully comprehensive exploratory screening for microRNA candidates associated with psychiatric comorbidities.

Therefore, the limitations which underly the exploratory character of the study design need to be taken into account.

## Conclusion

Data sets from the electrical post-SE model suggest different circulatory microRNA biomarker candidates for psychiatric comorbidities and for severity and distress in laboratory animals.

However, cross-model analysis argues against generalizability across different chronic epilepsy models. Thus, further research is necessary to compare the predictive validity of rodent epilepsy models for detection and management of psychiatric comorbidities.

## Material and methods

### Ethic statement

All animal experiments were conducted and reported in accordance with German law for animal protection and with the European Directive, 2010/63/EU. All animal experiments were approved by the government of Upper Bavaria (Munich, Germany, license number 55.2‐1‐54‐2531‐119‐14 and 55.2-1-54-2532-105-16). All experimental procedures have been carried out in compliance with the ARRIVE (Animal Research: Reporting of In Vivo Experiments) guidelines and the Basel declaration (http://www.basel.declaration.org) including the 3R principle.

### Animal models

Female Sprague Dawley rats (Envigo, Italy) were used to generate three rat models of epilepsy. The three rat epilepsy models were generated at the Institute of Pharmacology, Toxicology, and Pharmacy (Ludwig-Maximilians-University Munich, Germany): (1) the kindling model with repeated electrical induction of seizures; (2) the electrical, and (3) the chemical post-status epilepticus (SE) model with development of spontaneous seizures. The samples used in this study originated from three published studies, a study overview is provided in Supplementary Fig. 2. An extensive description of each of the three models used can be found in the respective publications: electrical post-SE model^18^, chemical post-SE model^17^, and kindling model^16^. In this study, subgroups of animals have been included: electrical post-SE model (exp n = 6, naïve n = 5, sham n = 5), chemical post-SE model (sham n = 12, exp n = 13), and kindling model (generalized sham n = 12, exp n = 11; focal sham/exp n = 12). The experimental group in the post-SE model comprised five randomly selected animals which had developed spontaneous recurrent seizures, and one additional animal which had developed high-frequency seizures to increase intragroup variability. The first part of the present study has an exploratory, hypothesis-generating character, therefore sample sizes could not be calculated. The second part was designed as a confirmatory and hypothesis-testing study and post hoc power was computed. For all models, female animals have been used. Please note that we used female rats based on the model characterization of Brandt and colleagues^44^ demonstrating a high mortality rate in male rats. Accordingly, female animals were also used in the other two models. All experimental (exp) and sham animals received an electrode implanted in either their right basolateral amygdala (AP-2.2 mm, L + 4.7 mm, DV + 8.5 mm, kindling and electrical post-SE models) or their right hippocampus (AP-3.9 mm, L + 1.7 mm, DV + 4.0/+ 4.1 mm, chemical post-SE model). The operative state was reached with multimodal anesthesia under the combination of chloral hydrate (360 mg/kg i.p.), inducing hypnosis and akinesia without affecting seizure thresholds, bupivacaine (0.5% up to 1 ml, Jenapharm, Germany, s.c.) for local anesthesia, and meloxicam (Metacam^®^, Boehringer Ingelheim, Germany, 1 mg/kg, 30 min pre- and 24 h post-surgery, s.c.) as perioperative analgesic. Details on group allocation, randomization, and the surgical procedures are provided in the Supplementary file. For the electrical post-SE model, an additional naïve, non-implanted control group (ctr) was included to determine potential effects of the chronic electrode implant. For all other models, electrode implanted, non-epileptic animal groups served as controls (sham).

### Blood sampling

For the microRNA expression analysis, plasma samples from the electrical post-SE model [naïve (n = 5), sham (n = 5), and experimental condition (n = 6)] were used. Blood samples were collected via cardiac puncture during endpoint-measurements under injection anesthesia [metamizole (100 mg/kg) and chloralhydrate (360 mg/kg)] followed by euthanasia (pentobarbital 600 mg/kg). To obtain liquid supernatant by separating the corpuscular components, total blood was centrifuged (2000*g* for 10 min), and plasma was stored in RNase/DNase clean tubes at − 80 °C till RNA-isolation.

### Behavioral and biochemical analysis

Behavioral and biochemical data from the electrical and chemical post-SE models and the kindling model were evaluated to identify and compare differences between the different chronic models as a means of validation. All animals were subjected to a comprehensive assessment of behavioral and biochemical parameters. The data from microRNA profiling and from behavioral and biochemical parameters originate from the same experimental and sham cohort. BDNF concentrations were measured in re-thawed serum samples using highly sensitive and specific fluorometric two-site enzyme-linked immunosorbent assays (ELISAs, Promega Inc, Germany) according to Hellweg and colleagues and Deuschle and colleagues^22,23^. A detailed description of the behavioral tests performed and the determination of BDNF serum concentrations can be found in the Supplementary file, the corresponding results for each model can be found in the respective publications^16–18^.

### RNA isolation from plasma samples

The miRNeasy plasma Mini Kit (Qiagen, Hilden, Germany) was utilized according to instructions for the RNA isolation. 30 μl of RNA-solution were obtained from an amount of 100 μl plasma. Synthetic Caenorhabditis elegans miR-39 (5 μl of 1 fmol/μl) was added as spike-in control during RNA isolation to the Qiazol/chloroform/plasma mixture.

### TaqMan ArrayCard microRNA screening

For the analysis of a microRNA expression profile, we selected the electrical post-SE model and a blinded and independent operator randomly selected five samples per group (sham and experimental condition), in addition one animal with a high number of seizures was included in the experimental group in order to increase the range concerning intrinsic disease severity. To consider a potential impact of the chronic electrode implant on microRNA expression, the sham group was selected as the control group for direct comparison with the rats with spontaneous recurrent seizures. After preamplification, TaqMan gene expression array cards—384-well microfluidic cards for rodents (Rodent A and B Card Set v3, ThermoFisher SCIENTIFIC, Darmstadt, Germany) were used according to manufacturer’s instructions. 750 different reactions of microRNAs highly conserved between mouse and rat plus 6 controls were measured.

### Statistical analysis

For array card analysis, p-values < 0.05 defined significance using a two-sided *t* test. Differential expression of microRNAs (Ct-values) was analyzed by Kruskal–Wallis test (Nonparametric One-way ANOVA multi-comparison test). Global normalization for qRT-PCR data was applied using the ddCT approach with the calculation of median values^27^. For correlation analysis of selected microRNAs with behavioral and biochemical parameters, Spearman correlation was calculated, and heatmaps were visualized using R version 3.6.1^54^ and the R package “corrplot”^55^. For correlation analysis of microRNA-429 with behavioral and biochemical parameters, a correlation coefficient r of < − 0.5 or > 0.5 in combination with p < 0.05 defined significant results. P values retrieved from Spearman correlation analysis of miR-429 were further corrected for multiple comparisons using false discovery rate (FDR) correction with the Benjamini–Hochberg method. Principal component analysis (PCA) was conducted using R version 4.1.2^54^, and graphical illustrations of the PCA were created with ggplot2^56^. Expression data were plotted as individual data points. Direct comparisons were computed by two-tailed unpaired *t* test. The significance level for all statistical tests performed was set at p < 0.05. For statistical analysis and graphical illustration of phenotypic and expression data, Graph Pad Prism 6 was utilized. The correlation matrix was visualized using R version 3.6.1^54^ and the R package “corrplot”^55^. Post-hoc power analyses were conducted using G*Power version 3.1.9.7; details on effect sizes and power calculation protocols are provided in the Supplementary file.

## Supplementary Information

Supplementary Information.

## Data Availability

The raw data of the current study are available in the Figshare repository 10.6084/m9.figshare.22270045.v1.

## References

[1] Thijs RD, Surges R, O'Brien TJ, Sander JW (2019). Epilepsy in adults. Lancet.

[2] Salpekar JA, Mula M (2019). Common psychiatric comorbidities in epilepsy: How big of a problem is it?. Epilepsy Behav..

[3] Scott AJ, Sharpe L, Loomes M, Gandy M (2020). Systematic review and meta-analysis of anxiety and depression in youth with epilepsy. J. Pediatr. Psychol..

[4] Snoeijen-Schouwenaars FM (2019). Mood, anxiety, and perceived quality of life in adults with epilepsy and intellectual disability. Acta Neurol. Scand..

[5] Pitkänen A (2016). Advances in the development of biomarkers for epilepsy. Lancet Neurol..

[6] Yazit NAA (2020). Association of micro RNA and postoperative cognitive dysfunction: A review. Mini Rev. Med. Chem..

[7] Biessels GJ, Nobili F, Teunissen CE, Simó R, Scheltens P (2020). Understanding multifactorial brain changes in type 2 diabetes: A biomarker perspective. Lancet Neurol..

[8] Aarsland D (2021). Parkinson disease-associated cognitive impairment. Nat. Rev. Dis. Primers.

[9] Pitkänen A, EkolleNdode-Ekane X, Lapinlampi N, Puhakka N (2019). Epilepsy biomarkers—Toward etiology and pathology specificity. Neurobiol. Dis..

[10] Pasquinelli AE (2000). Conservation of the sequence and temporal expression of let-7 heterochronic regulatory RNA. Nature.

[11] Hammond SM (2015). An overview of microRNAs. Adv. Drug Deliv. Rev..

[12] Minjarez B (2017). Behavioral changes in models of chemoconvulsant-induced epilepsy: A review. Neurosci. Biobehav. Rev..

[13] Sankar R, Mazarati A, Noebels JL (2012). Jasper's Basic Mechanisms of the Epilepsies.

[14] Bleich A, Tolba RH (2017). How can we assess their suffering? German research consortium aims at defining a severity assessment framework for laboratory animals. Lab. Anim..

[15] van Dijk RM (2020). Design of composite measure schemes for comparative severity assessment in animal-based neuroscience research: A case study focussed on rat epilepsy models. PLoS One.

[16] Möller C (2018). Toward evidence-based severity assessment in rat models with repeated seizures: I. Electrical kindling. Epilepsia.

[17] Koska I (2019). Toward evidence-based severity assessment in rat models with repeated seizures: II. Chemical post-status epilepticus model. Epilepsia.

[18] Seiffert I (2019). Toward evidence-based severity assessment in rat models with repeated seizures: III. Electrical post-status epilepticus model. Epilepsia.

[19] Boldt L (2021). Toward evidence-based severity assessment in mouse models with repeated seizures: I. Electrical kindling. Epilepsy Behav..

[20] Rana T, Behl T, Sehgal A, Srivastava P, Bungau S (2021). Unfolding the role of BDNF as a biomarker for treatment of depression. J. Mol. Neurosci..

[21] Szuhany KL, Otto MW (2020). Assessing BDNF as a mediator of the effects of exercise on depression. J. Psychiatr. Res..

[22] Deuschle M (2013). Changes of serum concentrations of brain-derived neurotrophic factor (BDNF) during treatment with venlafaxine and mirtazapine: Role of medication and response to treatment. Pharmacopsychiatry.

[23] Hellweg R, von Arnim CA, Büchner M, Huber R, Riepe MW (2003). Neuroprotection and neuronal dysfunction upon repetitive inhibition of oxidative phosphorylation. Exp. Neurol..

[24] Malhi GS, Mann JJ (2018). Depression. Lancet.

[25] Kumstel S (2020). MicroRNAs as systemic biomarkers to assess distress in animal models for gastrointestinal diseases. Sci. Rep..

[26] Qureshi R, Sacan A (2013). A novel method for the normalization of microRNA RT-PCR data. BMC Med. Genom..

[27] Livak KJ, Schmittgen TD (2001). Analysis of relative gene expression data using real-time quantitative PCR and the 2(−Delta Delta C(T)) method. Methods.

[28] Crawley J (2007). What's Wrong With My Mouse?.

[29] Klein S, Bankstahl JP, Löscher W, Bankstahl M (2015). Sucrose consumption test reveals pharmacoresistant depression-associated behavior in two mouse models of temporal lobe epilepsy. Exp. Neurol..

[30] Becker C (2015). Predicting and treating stress-induced vulnerability to epilepsy and depression. Ann. Neurol..

[31] Guo CM, Liu SQ, Sun MZ (2020). miR-429 as biomarker for diagnosis, treatment and prognosis of cancers and its potential action mechanisms: A systematic literature review. Neoplasma.

[32] Löscher W (2017). Animal models of seizures and epilepsy: Past, present, and future role for the discovery of antiseizure drugs. Neurochem. Res..

[33] Brandt C, Ebert U, Löscher W (2004). Epilepsy induced by extended amygdala-kindling in rats: Lack of clear association between development of spontaneous seizures and neuronal damage. Epilepsy Res..

[34] Möller C (2019). Impact of repeated kindled seizures on heart rate rhythms, heart rate variability, and locomotor activity in rats. Epilepsy Behav..

[35] Löscher W (2002). Animal models of epilepsy for the development of antiepileptogenic and disease-modifying drugs. A comparison of the pharmacology of kindling and post-status epilepticus models of temporal lobe epilepsy. Epilepsy Res..

[36] Müller CJ, Gröticke I, Bankstahl M, Löscher W (2009). Behavioral and cognitive alterations, spontaneous seizures, and neuropathology developing after a pilocarpine-induced status epilepticus in C57BL/6 mice. Exp. Neurol..

[37] Brindley E, Hill TDM, Henshall DC (2019). MicroRNAs as biomarkers and treatment targets in status epilepticus. Epilepsy Behav..

[38] Amin ND (2021). A hidden threshold in motor neuron gene networks revealed by modulation of miR-218 dose. Neuron.

[39] Tam S, Tsao M-S, McPherson JD (2015). Optimization of miRNA-seq data preprocessing. Brief. Bioinform..

[40] Mestdagh P (2009). A novel and universal method for microRNA RT-qPCR data normalization. Genome Biol..

[41] Liu C (2020). Altered response to total body irradiation of C57BL/6-Tg (CAG-EGFP) mice. Dose Response.

[42] Thapar A, Roland M, Harold G (2005). Do depression symptoms predict seizure frequency—or vice versa?. J. Psychosom. Res..

[43] Thompson NJ (2020). The impact of a depression self-management intervention on seizure activity. Epilepsy Behav..

[44] Brandt C, Glien M, Potschka H, Volk H, Löscher W (2003). Epileptogenesis and neuropathology after different types of status epilepticus induced by prolonged electrical stimulation of the basolateral amygdala in rats. Epilepsy Res..

[45] Clayton JA, Collins FS (2014). Policy: NIH to balance sex in cell and animal studies. Nature.

[46] McKinney WT (2001). Overview of the past contributions of animal models and their changing place in psychiatry. Semin. Clin. Neuropsychiatry.

[47] Schaffner KF, Peter M (2001). Theory and Method in the Neurosciences.

[48] Kafkafi N (2018). Reproducibility and replicability of rodent phenotyping in preclinical studies. Neurosci. Biobehav. Rev..

[49] Engel GL (1977). The need for a new medical model: A challenge for biomedicine. Science.

[50] Papadimitriou G (2017). The, "Biopsychosocial Model": 40 years of application in Psychiatry. Psychiatriki.

[51] Lemoine M, Wakefield JC, Demazeux S (2016). 2016. Sadness or Depression? International Perspectives on the Depression Epidemic and Its Meaning.

[52] Schaffner KF (2020). A comparison of two neurobiological models of fear and anxiety: A "construct validity" application?. Perspect. Psychol. Sci..

[53] Vervliet B, Raes F (2013). Criteria of validity in experimental psychopathology: Application to models of anxiety and depression. Psychol. Med..

[54] R Core Team. *R: A language and environment for statistical computing.* https://www.R-project.org/ (2020).

[55] Wei, T. & Simko, V. *R package *“*corrplot*”*: Visualization of a Correlation Matrix.* https://github.com/taiyun/corrplot (2019).

[56] Wickham, H. *ggplot2*: *Elegant Graphics for Data Analysis*. https://ggplot2.tidyverse.org (Springer, ISBN 978-3-319-24277-4, 2016).

