# Supplementary material for: An exploratory approach to identify microRNAs as circulatory biomarker candidates for epilepsy-associated psychiatric comorbidities in an electrical post-status epilepticus model
Source: Sci Rep. 2023 Mar 20;13:4552. doi: 10.1038/s41598-023-31017-9 (PMC10027890; doi:10.1038/s41598-023-31017-9)
Supplement: Supplementary file 1 — Supplementary Information. [file 41598_2023_31017_MOESM1_ESM.docx]

***Supplementary Information***

**An exploratory approach to identify microRNAs as circulatory biomarker candidates for epilepsy-associated psychiatric comorbidities in an electrical post-status epilepticus model**

Eva-Lotta von Rüden, Heike Janssen-Peters, Maria Reiber, Roelof Maarten van Dijk, Ke Xiao, Isabel Seiffert, Ines Koska, Christina Hubl, Thomas Thum, Heidrun Potschka.

**^Supplementary methods^**

1. **^Animal models^**
2. **^Behavioral and biochemical analysis^**
3. **^Post hoc power analysis^**

**^Supplementary figures 1-2^**

**^Supplementary tables 1-3^**

**^Supplementary references^**

***Supplementary methods***

Detailed descriptions of the animal models and for the analysis of behavioral and biochemical readouts have already been provided by our group in the context of a bioinformatic approach aiming to decipher top ranking parameters among the candidate parameters for severity assessment ^1^.

1. Animal models

For all models, female animals have been used. All experimental (exp) and sham animals received an electrode implanted in either their right basolateral amygdala (AP-2.2 mm, L+4.7 mm, DV+8.5 mm, kindling and electrical post-SE models) or their right hippocampus (AP-3.9 mm, L+1.7 mm, DV+4.0/+4.1 mm, chemical post-SE model). The bipolar, Teflon-isolated stainless-steel electrode was stereotactically implanted under general anesthesia (i.p. chloral hydrate 360 mg/kg) and multimodal analgesia with local anesthesia (s.c. bupivacaine 0.5% up to 1 ml, Jenapharm, Germany) and meloxicam as perioperative analgesic (Metacam®, Boehringer Ingelheim,Germany, 1 mg/kg, 30 min pre- and 24 h post-surgery, s.c.).

*1.1 Kindling model (for original publication, please see Ref.^2^):*

Three weeks following surgery the afterdischarge threshold (ADT) was determined for each animal. The kindling procedure was started two days following ADT determination. Five days a week, animals received a stimulation of 700 µA (1 ms, monophasic square wave pulses, 50 Hz for 1 s). Daily suprathreshold stimulations were continued until 10 generalized stage 5 seizures were reached.

Before the start of the study, a power analysis had been conducted in order to determine sample sizes using BIAS for Windows Version 10.04 software. In the original study, rats (n = 111) were randomly divided into two groups, comprising a group which received repeated stimulations once daily including a phase of generalized seizures (20 kindled rats, 18 electrode-implanted rats, and 18 naïve control rats), and a group receiving repeated stimulations with induction of focal seizures only (20 kindled rats, 18 electrode-implanted rats, and 17 naïve control rats). For the social interaction test, all animals were used, while for all other behavioral and biochemical tests, only 12 animals randomly selected from each subgroup were used. Group allocation was determined randomly (www.randomizer.org).

*1.2 Chemical post-SE model (for original publication, please see Ref.^3^):*

Three weeks following surgery, SE was induced by injection of pilocarpine. 14-16 h before pilocarpine treatment animals were injected with lithium chloride (i.p. 127 mg/kg, Sigma-Aldrich, Chemie GmbH, Taufkirchen, Germany), and 30 min before treatment, animals were injected with scopolamine methyl-bromide (i.p. 1 mg/kg, Sigma-Aldrich Chemie GmbH, Taufkirchen, Germany). Pilocarpine (10 mg/kg i.p., Sigma-Aldrich Chemie GmbH, Taufkirchen, Germany) was then injected every 30 min (maximum of 10 injections) until a SE was induced. SE was terminated by injections of phenobarbital (i.p. 25 mg/kg) and multiple injections of diazepam (i.p. 10 mg/kg, Ratiopharm; Ulm, Germany).

Before the start of the study, a power analysis had been conducted in order to determine sample sizes using BIAS for Windows Version 10.04 software. In the original study focusing on tethered recordings, rats (n = 44) were randomly divided into three groups, comprising a naïve (n = 12), a sham (n = 12) and a pilocarpine-treated tethered (n = 20) subgroup. Group allocations were determined randomly (www.randomizer.org). The number of animals used for behavioral and biochemical analysis varied within the study for several of the parameters.

*1.3 Electrical post-SE model (for original publication, please see Ref. ^4^)*:

Six weeks following surgery, SE was induced by a 25 min long stimulation of the basolateral amygdala with a 700 µA stimulation (intra-train pulse frequency of 50 Hz, 700 µA peak pulse intensity, 100 ms trains of 1 ms alternating positive and negative square-wave-pulses at a frequency of 2 Hz) resulting in a self-sustained SE. SE was terminated by injection of diazepam (i.p. 10 mg/kg, repeated dosing if necessary) four hours following the start of stimulation.

Before the start of the study, a power analysis had been conducted in order to determine sample sizes using BIAS for Windows Version 10.04 software. In the original study, focusing on tethered recordings, rats (n =44) were randomly divided into three groups, comprising a naïve (n = 12), a sham (n = 14) and an SE tethered (n = 18) subgroup. Group allocations were determined randomly (www.randomizer.org). The number of animals used for behavioral and biochemical analysis varied within the study for several of the parameters.

2. Behavioral and biochemical analysis

As described previously by our group ^1-4^, the analyses of nest building performance, latency to build nests, burrowing and saccharin preference were carried out in a home-cage based approach at the animal facility of the Institute of Pharmacology, LMU Munich. Open field, Black-white box and Elevated plus maze tests were conducted in a soundproof testing room at the Institute of Pharmacology, and these tests were video-recorded. For all of the behavioral experiments performed in a separate testing room, animals were allowed to habituate for at least 30 minutes to the experimental setting before the start of the experiment. The analysis of the videos was conducted with the video-tracking software EthoVision XT 8.5 (Noldus, Wageningen, the Netherlands). The investigator was familiar to the animals. The investigator was blinded for group allocation. Testing materials were cleaned with 0.1% acetic acid after each trial.

*2.1 Nest building and latency to nest building*

The assessment of nest building behavior was based on protocols developed by Van Loo and Baumans (2004) and Jirkof and colleagues (2013) ^5,6^. Firstly, continuous home-cage based video recording was applied to identify a peak of nest complexity during the circadian rhythm of the rats. Based on these data, pictures of the nests were taken at the peak of nest complexity, defined as between 7:00 and 9:00 a.m. The scoring comprised the assessment of the complexity of the nest as well as the shape of the nesting material (0 = not touched/ destroyed, 1 = flat, 2 = slightly curved, 3 = deep). Nesting behavior was investigated on a daily basis: 1.) before implantation in order to determine a baseline value, 2.) after the implantation of the stimulation electrode, as well as 3.) during phases with repeated focal or generalized seizures. Complexity and shape of the nest were assessed on five consecutive days during the kindling phase with generalized seizures (days of 9th to 13th generalized seizure). Animals received new nesting material after four days. The latency to nest building was assessed in a new cage with 14 g of fresh embedding material (Enviro-dri, Claus GmbH, Limburgerhof, Germany) once a week. We defined nest building activity as digging into, nibbling on or carrying around nesting material for more than three seconds. Directly after the offer of new nesting material, animals were observed for ten minutes, and the latency time for the start of nest building activity was determined in seconds. In cases where no nest building activity was observed within 10 minutes, a value of 600 seconds was used for data analysis.

Following a protocol by Van Loo and Baumans (2004) ^5^, we assessed the level of soiling (1 = clean, 2 = slightly soiled, 3 = substantially soiled) once a week before the animals received fresh embedding material.

*2.2 Burrowing*

The burrowing test was conducted following a protocol by Rutten and colleagues (2014) ^7,8^. In short: each animal was placed in a Makrolon Type IV cage with a plastic burrowing tube (32 cm long x 10 cm Ø, elevated on one site 6 cm) filled with 2.5 kg gravel (quartz-light, grain size 2 - 4 mm, ORBIT GmbH, Germany). Prior to the collection of baselines (before surgery) and experimental data, all animals had a training phase comprising four days: on the first day, animals received an empty burrowing tube for 60 minutes; on the following three days, animals received a gravel-filled tube for 60 minutes after a habituation time of 30 minutes in the empty cage. Burrowing performance was assessed on day 5 for baseline values, followed by assessments one week after surgery, and after repeated generalized seizure. The latency to start burrowing activity (seconds) was measured directly after the burrowing tube had been placed into the cage. After the total observation time, the tube with the remaining gravel was weighed to determine the amount of gravel burrowed by the rats.

Two animals with a baseline burrowing performance less than 500 g were excluded from the data analysis of burrowing activity.

*2.3 Social Interaction Test*

The social interaction test was performed following a protocol by Hölter and colleagues (2015) and File and colleagues (2004) ^9,10^: At least two weeks prior to the social interaction test, rats were housed individually in order to prevent contact between the animals and to maximize the time rats will spend in social interaction. On two consecutive days, the rats habituated to the experimental setting. Therefore, the animals were transferred to the behavioral room (lighting condition: 20 lux), and placed into Makrolon Type IV cages (one animal per cage) for ten minutes. For the experiment, two weight-matched rats were placed together into the testing arena. We recorded the duration rats spent in active social interaction (parameters scored: sniffing, grooming, play, following, walking on each other). For each pair, a combined score was obtained.

*2.4 Saccharin preference test*

The saccharin preference test was conducted following a protocol by Klein and colleagues (2015) ^11^. During the four consecutive days of testing, animals received two water bottles (total volume per bottle: 700 ml; 70 x 70 mm square; Ehret Labor- und Pharmatechnik, Germany) in order to avoid side preferences. On the first day and on the third day, both bottles were filled with 500 g water to determine the water intake of the animals over 24 hours. On the second and fourth day, one of the two bottles was filled with 500 g of a 0.1% saccharin solution (Aldrich Saccharin ≥ 98%, Sigma-Aldrich Chemie GmbH, Germany), while the other bottle contained 500 g water. On the second and fourth day, the side of the bottle containing the saccharin solution was alternated from the left to the right in order to avoid side preferences.

The open field, black-white box, and elevated plus maze tests were carried out as described previously ^1,12^, in short:

*2.5 Open field test*

The open field test was applied to investigate locomotor activity and exploratory behavior. Animals were placed individually in a round open field (Ø = 85 cm, 10-20 lux), 10 cm apart from and facing the arena wall. The total test and tracking duration lasted 10 minutes.

The following behavioral parameters were among the readouts assessed with the tracking software EthoVision XT 8.5 (Noldus): the total distance moved by the animals, immobility time, frequency of the posture ‘rearing’, and the time rats spent in each zone of the arena (wall and center).

*2.6 Black-white box*

The experimental set-up of the black-white box comprises a plastic box with a black compartment (39 x 20 x 39 cm, with a top) and a white compartment (39 x 39 x 39 cm, 50 lux), connected via a poorly illuminated tunnel. For the experiment, the animal was placed into the white compartment with its head facing the tunnel. The test duration was five minutes. The following behavioral parameters were among the readouts assessed with the tracking software EthoVision XT 8.5 (Noldus): latency to enter the black box, stretching postures, time spent in the black and the white compartment.

*2.7 Elevated plus maze*

The elevated plus maze, as described previously by File and colleagues (1993) ^13^, was applied as a behavioral assay to score anxiety-related readouts. The experimental set-up comprises a central platform (14 x 14 cm), two open arms (50 x 14 cm, 20 lux), and two closed arms (15 x 14 x 29 cm, 10 lux), elevated 82 cm above the ground. For the conduction of the test, the animal was placed into the center of the maze facing the same closed arm. The total test duration was five minutes. The tracking was performed using the software EthoVision XT8.5 (Noldus).

*2.8 Serum analysis*

The collection of blood samples represents an invasive means, it was carried out at the end of the experiments in order to reduce its possible impact on the candidate parameters of severity assessment. Prior to blood sampling, animals could habituate to the experimental setting for at least 30 minutes. Blood sampling was carried out as follows: 36 hours after the last seizure, retrobulbar puncture was done under isoflurane anesthesia in the morning (9:00 to 10:30 a.m.). Eppendorf vials containing the blood were left for 30 minutes. After coagulation, the blood was centrifuged at 1500 x g for 10 minutes, and samples were stored frozen at -80°C.

BDNF concentrations were measured in re-thawed serum samples (1:20 diluted with sample buffer) using highly sensitive and specific fluorometric two-site enzyme-linked immunosorbent assays (ELISAs). The ELISAs were applied in accordance with the manufacturer’s instructions (Promega Inc, Germany), and modified in the following manner: primary anti-BDNF monoclonal antibody, anti-human-BDNF polyclonal antibody, and goat anti-chicken-IgY-alkaline phosphatase polyclonal secondary antibody were allocated exclusively for the procedure. An overview of the detailed procedure has been provided by Hellweg and colleagues (2003) and Deuschle and colleagues (2013) ^14,15^.

1. *Post hoc power analysis*
   1. *Power analysis across models:*

Effect size:

Cohens’s d: (4.769 - 3.984) / 1.749378 = 0.464165

Based on Sham: Mean: 3.984 Exp: 4.769

Standard deviation: 1.912 1.57

Sample size: 42 40

Protocol power calculation:

**t tests -** Means: Difference between two independent means (two groups)

**Analysis:** Post hoc: Compute achieved power

**Input:** Tail(s) = One

Effect size d = 0.464165

α err prob = 0.05

Sample size group 1 = 42

Sample size group 2 = 40

**Output:** Noncentrality parameter δ = 2.1009712

Critical t = 1.6641246

Df = 80

Power (1-β err prob) = 0.6694148

- 1. *Power analysis kindling model – focal seizures:*

Effect size:

Cohens’s d: (3.992 – 2.586) / 1.77974 = 0.800117

Based on Sham: Mean: 2.568 Exp: 3.992

Standard deviation: 2.074 1.426

Sample size: 12 12

Protocol power calculation:

**t tests -** Means: Difference between two independent means (two groups)

**Analysis:** Post hoc: Compute achieved power

**Input:** Tail(s) = One

Effect size d = 0.800117

α err prob = 0.05

Sample size group 1 = 12

Sample size group 2 = 12

**Output:** Noncentrality parameter δ = 1.9598784

Critical t = 1.7171444

Df = 22

Power (1-β err prob) = 0.6003418

- 1. *Power analysis kindling model – generalized seizures:*

Effect size:

Cohens’s d: (4.202 – 3.405) / 1.52906 = 0.521235

Based on Sham: Mean: 3.405 Exp: 4.202

Standard deviation: 1.659 1.387

Sample size: 12 11

Protocol power calculation:

**t tests -** Means: Difference between two independent means (two groups)

**Analysis:** Post hoc: Compute achieved power

**Input:** Tail(s) = One

Effect size d = 0.521235

α err prob = 0.05

Sample size group 1 = 12

Sample size group 2 = 11

**Output:** Noncentrality parameter δ = 1.2486957

Critical t = 1.7207429

Df = 21

Power (1-β err prob) = 0.3312793

- 1. *Power analysis chemical post-SE model:*

Effect size:

Cohens’s d: (6.118 – 5.135) / 1.228966 = 0.79986

Based on Sham: Mean: 5.135 Exp: 6.118

Standard deviation: 1.413 1.012

Sample size: 12 13

Protocol power calculation:

**t tests -** Means: Difference between two independent means (two groups)

**Analysis:** Post hoc: Compute achieved power

**Input:** Tail(s) = One

Effect size d = 0.79986

α err prob = 0.05

Sample size group 1 = 12

Sample size group 2 = 13

**Output:** Noncentrality parameter δ = 1.9980496

Critical t = 1.7138715

Df = 23

Power (1-β err prob) = 0.6155856

***Supplementary figures 1-2***

Supplementary figure 1: Flow diagram of the applied microRNA filtering and selection process. Created with BioRender.com.


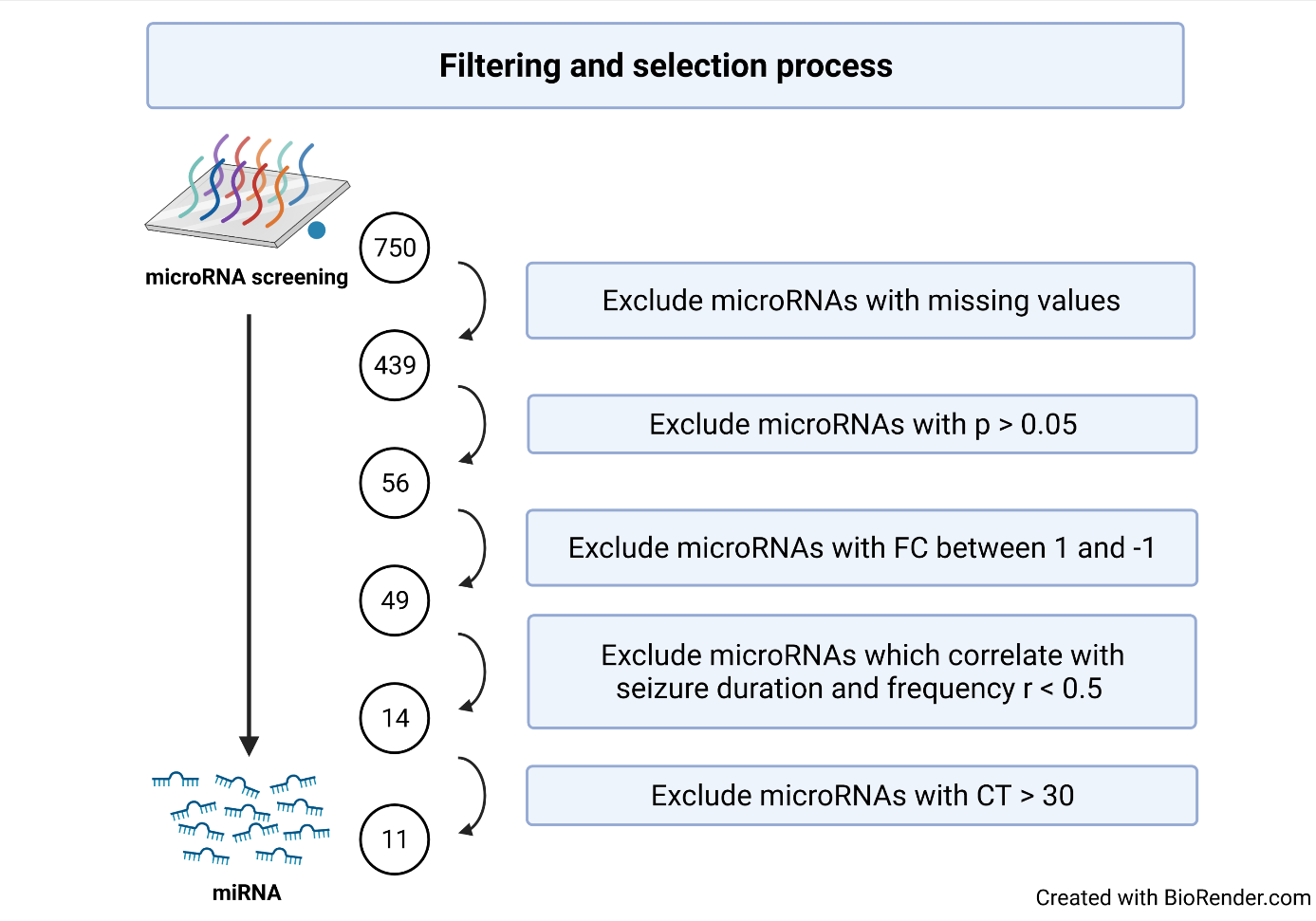


Supplementary figure 2: Timeline of the experiments


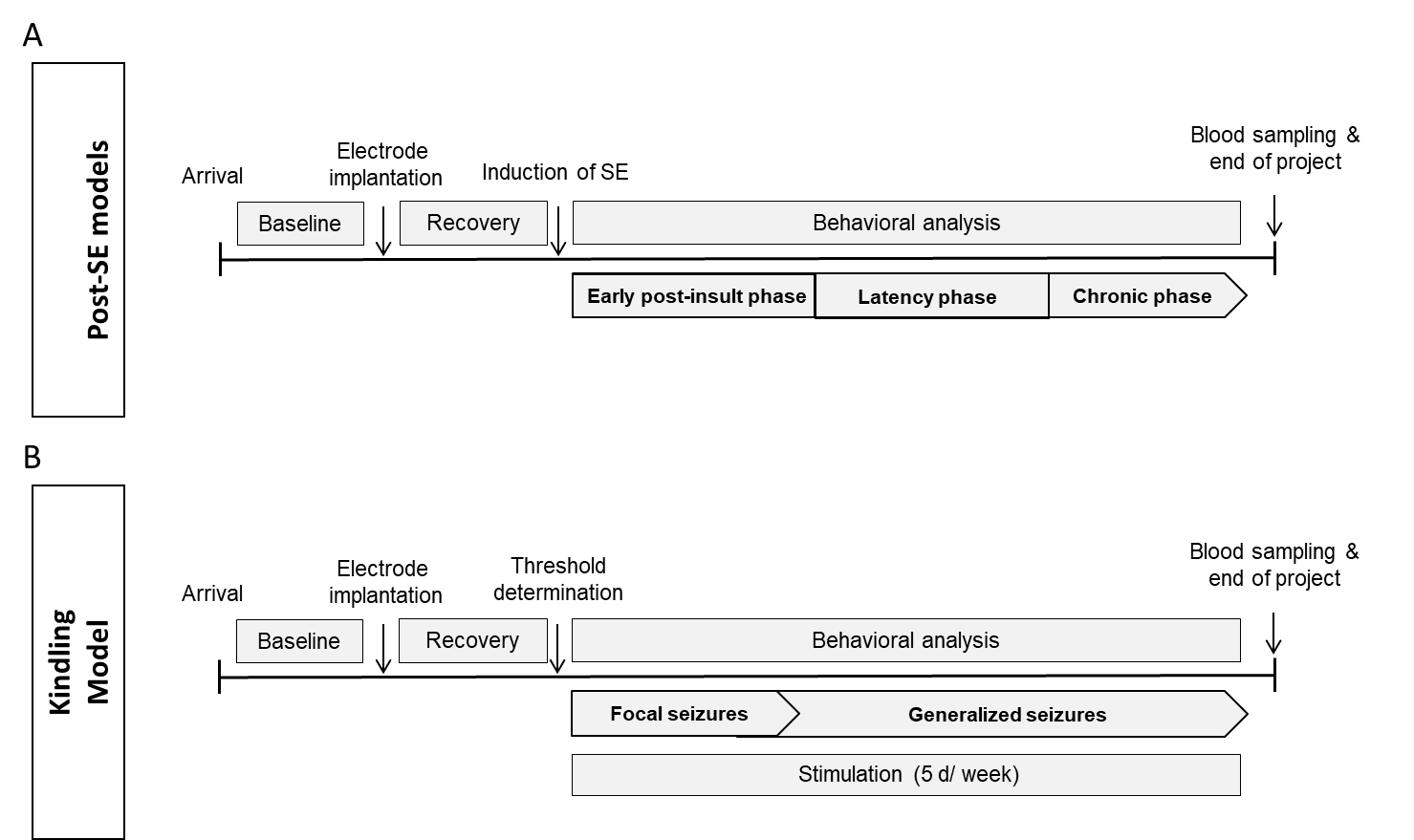


Timeline of the experiments according to Möller et al. ^2^, Koska et al. ^3^, and Seiffert, et al.^4^. To capture changes in distress or health related modifications the bodyweight of all animals was measured daily. In addition, a modified Irwin score and the Grimace scale were assessed. Baselines for burrowing and nest building behavior were analyzed before electrode implantation and during the recovery period in all experiments. At the end of the experiments, animals were sacrificed using pentobarbital (600 mg/kg i.p., Narcoren, Merial GmbH, Hallbergmoos, Germany), and blood samples were obtained for further analysis.

**A** Electrical and chemical post-SE models. The following time points were analyzed in these projects: the post-surgery recovery phase, the early post-insult phase, the latency phase, and the chronic phase following epilepsy manifestation.

**B** Kindling model: Animals received daily kindling stimulation. In the group with generalized seizures stimulations were continued until 14-15 generalized seizures (stage 4-5, 21 days) were elicited. In the groups with focal seizures, all animals received three stimulations inducing focal seizures (stage 1-3).

***Supplementary tables 1-3***

| Parameter | Test | Meaning |
| --- | --- | --- |
| Weight_gain | Weight gain | Animals were weighted on the first and the last day of the experiments and the difference was calculated in gram. |
| NB | Nestbuilding | Nestbuilding activity on the day of the 10^th^ generalized seizure (kindling model); with spontaneous seizure activity (week 11 post SE, post SE-models) |
| BUR | Burrowing | Burrowing behavior in the phase of generalized seizures. The displaced gravel was calculated in Gramm |
| SI | Social interaction | The time spent in active social interaction (sniffing, grooming, play, following, walking on each other) was recorded in seconds. |
| SP_percentage | Saccharin Preference | Saccharin preference was tested on 2 days compared to water. The total consumed amount was calculated and the percentage of saccharin intake was shown in percentage. |
| OF_distance | Open Field | Open Field distance moved total in centimeter. |
| OF_rearing | Open Field | Number of rearing postures in the Open Field. Rearing was defined as vertical activity with the animal rising more than 45% |
| OF_immobility | Open Field | Time in seconds the animal was immobile in the Open Field. |
| OF_center | Open Field | Time in seconds the animals spent in the center region of the Open Field |
| BWB_WB | Black and White Box | Time in seconds the animals spent in the white box. The time the animals spent in the black box was also measured, but is not shown here. |
| BWB_entries | Black and White Box | Number of transitions into the white compartment, |
| BWB_stretching | Black and White Box | Number of stretching postures of the animal in the Black and White Box. Stretching was defined as the animal looking out of the black into the white box with its body elongated. |
| BWB_LT | Black and White Box | Latency to the first entry of the black box from the white box in which the animals were placed. |
| EPM_streching | Elevated Plus Maze | Number of stretching postures of the animal in the Elevated Plus Maze. Stretching was defined as the animal looking out of one of the closed arms into an open arm. |
| EPM_head_dip | Elevated Plus Maze | Number of the times the animal looked down from one of the open arms. |
| EPM_closedarms | Elevated Plus Maze | Time the animal spent in both closed arms of the Elevated Plus Maze. |
| EPM_openarms | Elevated Plus Maze | Time the animal spent in both open arms of the Elevated Plus Maze. |
| EPM_open1_3 | Elevated Plus Maze | Time the animal spent in the outer 1/3 of the both open arms. |
| BDNF | BDNF | Brain-derived neurotrophic factor results are shown in pg/ml. |
| Adrenal_glands | Adrenal glands | The weight of both adrenal glands was calculated in gram. |

Supplementary table 1: Description of parameters used for the correlation matrix.

Supplementary table 2: Correlation values (p-value **(A)** and correlation coefficient r **(B)**) of the selected microRNAs and behavioral and biochemical parameters.


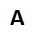


| p value | Weight_gain | NB | BUR | SI | SP_percentage | OF_distance | OF_rearing | OF_immobile | OF_center | BWB_WB | BWB_entries | BWB_stretching | BWB_LT | EPM_stretching | EPM_head_dip | EPM_closedarms | EPM_openarms | EPM_open1_3 | Adrenal_glands | BDNF | Seizures_n | Seizures_duration |
| --- | --- | --- | --- | --- | --- | --- | --- | --- | --- | --- | --- | --- | --- | --- | --- | --- | --- | --- | --- | --- | --- | --- |
| miR-376a | 0.003 | 0.538 | 0.244 | 0.01 | 0.001 | 0.067 | 0.01 | 0.168 | 0.957 | 0.494 | 0.260 | 0.617 | 1 | 0.237 | 0.546 | 0.187 | 0.231 | 0.257 | 0.642 | 0.071 | 0.553 | 0.624 |
| miR-429 | 0.011 | 0.708 | 0.105 | 0.009 | 0.002 | 0.119 | 0.273 | 0,163 | 0.753 | 0.049 | 0.218 | 0.806 | 0.829 | 0.275 | 0.884 | 0.454 | 0.603 | 0.593 | 0.528 | 0.01 | 0.913 | 0.329 |
| miR-494 | 0.001 | 0.844 | 0.317 | 0.001 | 0.025 | 0.156 | 0.273 | 0,965 | 0.55 | 0.376 | 0.319 | 0.482 | 0.483 | 0.404 | 0.976 | 0.37 | 0.563 | 0.522 | 0.919 | 0.028 | 0.957 | 0.329 |
| miR-763 | 0.002 | 0.763 | 0.191 | 0.014 | 0.026 | 0.015 | 0.221 | 0.378 | 0.897 | 0.124 | 0.701 | 0.879 | 0.812 | 0.463 | 0.679 | 0.936 | 0.979 | 1 | 0.633 | 0.031 | 0.658 | 0.957 |
| miR-342-3p | 0.173 | 0.545 | 0.892 | 0.36 | 0.713 | 0.641 | 0.513 | 0.974 | 0.405 | 0.633 | 0.329 | 0.085 | 0.704 | 0.057 | 0.097 | 0.116 | 0.14 | 0.113 | 0.039 | 0.345 | 0.321 | 0.704 |
| miR-1903 | 0.021 | 0.116 | 0.254 | 0.014 | 0.064 | 0.409 | 0.567 | 0.464 | 0.533 | 0.409 | 0.700 | 0.275 | 0.864 | 0.651 | 0.362 | 0.674 | 0.694 | 0.628 | 0.154 | 0.233 | 0.538 | 0.957 |
| miR-697 | 0.019 | 0.968 | 0.290 | 0.005 | 0.001 | 0.318 | 0.404 | 0.267 | 0.401 | 0.185 | 0.845 | 0.892 | 0.401 | 0.554 | 0.409 | 0.879 | 0.748 | 0.747 | 0.923 | 0.023 | 0.700 | 0.787 |
| miR-148b-5p | 0.104 | 0.168 | 0.374 | 0.068 | 0.048 | 0.18 | 0.414 | 0.644 | 0.208 | 0.176 | 0.482 | 0.78 | 0.729 | 0.769 | 0.247 | 0.625 | 0.541 | 0.523 | 0.983 | 0.036 | 0.658 | 0.544 |
| miR-712 | 0.095 | 0.939 | 0.207 | 0.009 | 0.03 | 0.322 | 0.605 | 0.897 | 0.871 | 0.102 | 0.658 | 0.546 | 0.641 | 0.458 | 0.301 | 0.835 | 0.639 | 0.642 | 0.244 | 0.009 | 0.354 | 0.957 |
| miR-203 | 0.118 | 0.923 | 0.991 | 0.069 | 0.132 | 0.076 | 0.447 | 0.392 | 0.04 | 0.144 | 0.198 | 0.56 | 0.154 | 0.769 | 0.611 | 0.718 | 0.95 | 0.934 | 0.641 | 0.478 | 0.461 | 0.872 |
| miR-598 | 0.979 | 0.988 | 0.845 | 0.508 | 0.502 | 0.391 | 0.602 | 0.665 | 0.812 | 0.009 | 0.188 | 0.886 | 0.217 | 0.133 | 0.6 | 0.852 | 0.873 | 0.873 | 0.983 | 0.255 | 0.741 | 0.873 |
|  | p<0.05 |  |  |  |  |  |  |  |  |  |  |  |  |  |  |  |  |  |  |  |  |  |

| 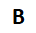 |  |  |  |  |  |  |  |  |  |  |  |  |  |  |  |  |  |  |  |  |  |  |
| --- | --- | --- | --- | --- | --- | --- | --- | --- | --- | --- | --- | --- | --- | --- | --- | --- | --- | --- | --- | --- | --- | --- |
| correlation coefficient | Weight_gain | NB | BUR | SI | SP_percentage | OF_distance | OF_rearing | OF_immobile | OF_center | BWB_WB | BWB_entries | BWB_stretching | BWB_LT | EPM_stretching | EPM_head_dip | EPM_closedarms | EPM_openarms | EPM_open1_3 | Adrenal_glands | BDNF | Seizures_n | Seizures_duration |
| miR-376a | 0.753 | 0,188 | -0.348 | -0.71 | -0.824 | 0.522 | 0.680 | -0.407 | -0.016 | 0.209 | 0.337 | 0.153 | 0 | -0.353 | 0.185 | 0.391 | 0.357 | 0.339 | 0.143 | -0.516 | -0.359 | -0.3 |
| miR-429 | 0.632 | -0.105 | -0.421 | -0.649 | -0.739 | 0.406 | 0.292 | -0.366 | -0.085 | 0.5 | 0.326 | 0.067 | -0.059 | -0.301 | -0.041 | 0.209 | 0.146 | 0.15 | -0.171 | -0.624 | -0.058 | 0.486 |
| miR-494 | 0.776 | 0.058 | -0.277 | -0.817 | -0.596 | 0.386 | 0.302 | -0.013 | 0.168 | 0.246 | 0.276 | 0.197 | -0.196 | -0.243 | -0.009 | 0.26 | 0.169 | 0.187 | -0.029 | -0.564 | 0.029 | 0.486 |
| miR-763 | 0.827 | -0.103 | -0.406 | -0.712 | -0.664 | 0.678 | 0.382 | -0.28 | -0.042 | 0.469 | 0.124 | -0.049 | -0.077 | -0.248 | -0.141 | 0.027 | -0.009 | 0 | -0.154 | -0.622 | 0.232 | 0.029 |
| miR-342-3p | -0.371 | -0.17 | -0.037 | 0.254 | 0,104 | 0.126 | 0.177 | 0.009 | -0.224 | -0.129 | 0.261 | 0.444 | 0.103 | -0.502 | 0.444 | 0.424 | 0.4 | 0.426 | 0.521 | 0.253 | -0.493 | -0.2 |
| miR-1903 | 0.632 | 0.457 | -0.327 | -0.662 | -0.527 | 0.24 | 0.167 | -0.213 | -0.182 | 0.24 | -0.113 | -0.314 | -0.051 | 0.139 | -0.275 | -0.129 | -0.121 | -0.149 | -0.402 | -0.341 | 0.319 | 0.029 |
| miR-697 | 0.637 | -0.012 | -0.304 | -0.725 | -0.813 | 0.288 | 0.242 | -0.319 | -0.244 | 0.376 | 0.057 | 0.04 | 0.244 | -0.181 | -0.251 | -0.047 | -0.099 | -0.099 | -0.029 | -0.6 | 0.203 | 0.143 |
| miR-148b-5p | 0.436 | 0.375 | -0.239 | -0.484 | -0.518 | 0.353 | 0.219 | -0.125 | -0.332 | 0.356 | 0.19 | -0.076 | -0.094 | -0.083 | -0.319 | -0.138 | -0.171 | -0.179 | 0.006 | -0.526 | -0.232 | -0.314 |
| miR-712 | 0.446 | 0.021 | -0.333 | -0.647 | -0.561 | 0.265 | 0.14 | -0.035 | 0.044 | 0.424 | 0.12 | -0.163 | -0.126 | -0.208 | -0.286 | -0.059 | -0.132 | -0.131 | -0.309 | -0.632 | 0.464 | -0.029 |
| miR-203 | 0.421 | -0.027 | -0.003 | -0.482 | -0.407 | 0.456 | 0.205 | -0.23 | 0.518 | 0.382 | 0.34 | 0.157 | -0.374 | -0.083 | -0.143 | 0.102 | 0.018 | 0.023 | -0.126 | -0.191 | -0.377 | 0.086 |
| miR-598 | -0.009 | 0.005 | 0.063 | 0.224 | 0.227 | -0.273 | 0.168 | 0.14 | -0.077 | -0.713 | -0.408 | 0.046 | 0.385 | 0.483 | 0.178 | 0.064 | 0.055 | 0.055 | -0.007 | 0.357 | 0.205 | 0.1 |
|  | r>0,5 | r<-0,5 |  |  |  |  |  |  |  |  |  |  |  |  |  |  |  |  |  |  |  |  |

Supplementary table 3: Correlation values (FDR-corrected p-value and correlation coefficient r) of the microRNA miR-429 and behavioral and biochemical parameters.

| p value | miR-429 | Weight_gain | NB | BUR | SI | SP_percentage | OF_distance | OF_rearing | OF_immobile | OF_center_time | BWB_WB | BWB_entries | BWB_streching | BWB_LT | EPM_stretching | EPM_head_dip | EPM_closedarms | EPM_openarms | EPM_open1_3 | Adrenal_glands | BDNF |
| --- | --- | --- | --- | --- | --- | --- | --- | --- | --- | --- | --- | --- | --- | --- | --- | --- | --- | --- | --- | --- | --- |
| miR-429 | NA | 0.2 | 0.8 | 0.1 | 0.1 | 0.5 | 0.01 | 0.2 | 0.2 | 0.2 | 0.5 | 0.8 | 0.6 | 0.2 | 0.02 | 0.3 | 0.1 | 0.2 | 0.2 | 0.9 | 0.01 |
|  | p<0.05 |  |  |  |  |  |  |  |  |  |  |  |  |  |  |  |  |  |  |  |  |
|  |  |  |  |  |  |  |  |  |  |  |  |  |  |  |  |  |  |  |  |  |  |
| correlation coefficient | miR-429 | Weight_gain | NB | BUR | SI | SP_percentage | OF_distance | OF_rearing | OF_immobile | OF_center_time | BWB_WB | BWB_entries | BWB_streching | BWB_LT | EPM_stretching | EPM_head_dip | EPM_closedarms | EPM_openarms | EPM_open1_3 | Adrenal_glands | BDNF |
| miR-429 | 1 | 0.06 | 0.01 | -0.02 | -0.12 | 0.00 | 0.23 | 0.34 | -0.08 | -0.15 | -0.13 | -0.07 | 0.10 | 0.23 | -0.41 | 0.32 | -0.36 | 0.19 | 0.28 | -0.34 | 0.570 |
|  | r>0.5 | r<-0.5 |  |  |  |  |  |  |  |  |  |  |  |  |  |  |  |  |  |  |  |

***Supplementary references***

1 van Dijk, R. M. *et al.* Design of composite measure schemes for comparative severity assessment in animal-based neuroscience research: A case study focussed on rat epilepsy models. *PLoS One* **15**, e0230141, doi:10.1371/journal.pone.0230141 (2020).

2 Möller, C. *et al.* Toward evidence-based severity assessment in rat models with repeated seizures: I. Electrical kindling. *Epilepsia* **59**, 765-777, doi:10.1111/epi.14028 (2018).

3 Koska, I. *et al.* Toward evidence-based severity assessment in rat models with repeated seizures: II. Chemical post-status epilepticus model. *Epilepsia* **60**, 2114-2127, doi:10.1111/epi.16330 (2019).

4 Seiffert, I. *et al.* Toward evidence-based severity assessment in rat models with repeated seizures: III. Electrical post-status epilepticus model. *Epilepsia* **60**, 1539-1551, doi:10.1111/epi.16095 (2019).

5 Van Loo, P. L. & Baumans, V. The importance of learning young: the use of nesting material in laboratory rats. *Lab Anim* **38**, 17-24, doi:10.1258/00236770460734353 (2004).

6 Jirkof, P. *et al.* Assessment of postsurgical distress and pain in laboratory mice by nest complexity scoring. *Lab Anim* **47**, 153-161, doi:10.1177/0023677213475603 (2013).

7 Rutten, K. *et al.* Burrowing as a non-reflex behavioural readout for analgesic action in a rat model of sub-chronic knee joint inflammation. *Eur J Pain* **18**, 204-212, doi:10.1002/j.1532-2149.2013.00358.x (2014).

8 Rutten, K., Robens, A., Read, S. J. & Christoph, T. Pharmacological validation of a refined burrowing paradigm for prediction of analgesic efficacy in a rat model of sub-chronic knee joint inflammation. *Eur J Pain* **18**, 213-222, doi:10.1002/j.1532-2149.2013.00359.x (2014).

9 Hölter, S. M. *et al.* Tests for Anxiety-Related Behavior in Mice. *Curr Protoc Mouse Biol* **5**, 291-309, doi:10.1002/9780470942390.mo150010 (2015).

10 File, S. E., Lippa, A. S., Beer, B. & Lippa, M. T. Animal tests of anxiety. *Curr Protoc Neurosci* **Chapter 8**, Unit 8.3, doi:10.1002/0471142301.ns0803s26 (2004).

11 Klein, S., Bankstahl, J. P., Löscher, W. & Bankstahl, M. Sucrose consumption test reveals pharmacoresistant depression-associated behavior in two mouse models of temporal lobe epilepsy. *Exp Neurol* **263**, 263-271, doi:10.1016/j.expneurol.2014.09.004 (2015).

12 Pekcec, A., Mühlenhoff, M., Gerardy-Schahn, R. & Potschka, H. Impact of the PSA-NCAM system on pathophysiology in a chronic rodent model of temporal lobe epilepsy. *Neurobiol Dis* **27**, 54-66, doi:10.1016/j.nbd.2007.04.002 (2007).

13 File, S. E. The interplay of learning and anxiety in the elevated plus-maze. *Behav Brain Res* **58**, 199-202, doi:10.1016/0166-4328(93)90103-w (1993).

14 Hellweg, R., von Arnim, C. A., Büchner, M., Huber, R. & Riepe, M. W. Neuroprotection and neuronal dysfunction upon repetitive inhibition of oxidative phosphorylation. *Exp Neurol* **183**, 346-354, doi:10.1016/s0014-4886(03)00127-4 (2003).

15 Deuschle, M. *et al.* Changes of serum concentrations of brain-derived neurotrophic factor (BDNF) during treatment with venlafaxine and mirtazapine: role of medication and response to treatment. *Pharmacopsychiatry* **46**, 54-58, doi:10.1055/s-0032-1321908 (2013).
